# Exploring the Child Functioning Module in Multiple Surveys in Ghana and Niger

**DOI:** 10.3390/ijerph23070892

**Published:** 2026-07-10

**Authors:** Arne H. Eide, Huafeng Zhang, Evelyn S. Adjei, Yacouba Daouda, Anthony K. Edusei, Aicha S. Goza, Mitch E. Loeb, Anne Hatløy

**Affiliations:** 1 Department of Health Research, Sintef Digital, PB 124 Blindern, 0373 Oslo, Norway; mitchloeb@yahoo.com; 2 Fafo Institute for Labour and Social Research, P.O. Box 2947 Tøyen, 0608 Oslo, Norway; huafeng.zhang@fafo.no (H.Z.); anne.hatloey@fafo.no (A.H.); 3 Department of Health Promotion and Disability Studies, School of Public Health, Kwame Nkrumah University of Science and Technology, Private Mail Bag, University Post Office, Kumasi, Ghana; enoserwaagh@yahoo.com (E.S.A.); akedusei.chs@knust.edu.gh (A.K.E.); 4 Departement des Science de l’Éducation, Faculté des Lettres et Sciences Humaines, École Normale Supérieure, Université Abdou-Moumouni, Niamey PB 10896, Niger; yacouba.bouwey@uam.edu.ne (Y.D.); nanaaicha.sandi@uam.edu.ne (A.S.G.)

**Keywords:** children, Child Functioning Module, measurement stability

## Abstract

**Highlights:**

**Public health relevance—How does this work relate to a public health issue?**

- Inclusive education fosters academic, social and emotional growth among children who are at risk of not accessing education, dropping out or face limited achievement in school.
- The study engages with measurement of disability among school children which is of importance for supporting and monitoring achievement of the SDGs and to implement the CRPD.

**Public health significance—Why is this work of significance to public health?**

- Proper assessment of disability among school children is a prerequisite for inclusive education.
- Assessment of disability among school children is an important step towards proper adaptation of the learning environment with implications for learning outcome, health and quality of life for children with functional difficulties.

**Public health implications—What are the key implications or messages for practitioners, policy makers and/or researchers in public health?**

- Systematic disability assessment in school is recommended to support proper adaptation of the learning environment.
- The Child Functioning Module is recommended for the assessment of functional difficulties among school children.

**Abstract:**

The Washington Group/UNICEF Child Functioning Module (CFM) represents progress in identifying and measuring disability among children and adolescents in censuses and surveys. This paper reports from a study among school children in Ghana and Niger aimed at investigating the stability in assessment using CFM over time and across different respondents. Four surveys and one mapping exercise using teachers and households/parents as proxies were implemented among primary school children in Ghana and Niger in 2022–2023. The surveys included CFM and variables intended to generate new knowledge about school achievement among children with disabilities. The results demonstrate considerable variation in disability classification across time and between respondent groups. Observed assessment consistency among and between teachers and parents varied largely between 60 and 70%, implying a discrepancy in disability status assessment of 30–40% over time and between respondent groups. Agreement was further analyzed using Prevalence-Adjusted and Bias-Adjusted Kappa (PABAK), yielding improved agreement as compared to Cohen’s Kappa. The study supports that CFM is useful as a survey instrument for identifying patterns of functional difficulties in children at the group or population level, but also that multiple respondents are recommended when proxy reporting is used. Further studies are needed in different contexts to explore CFM statistical properties and to reach a stronger evidence base on discrepancies in disability assessment over time and across different groups of respondents.

## 1. Introduction

The International Classification of Functioning, Disability and Health (ICF) [1] provides a common framework for conceptualizing disability that has stimulated development of a new generation of disability statistics [2]. Based on the ICF, the Washington Group on Disability Statistics (WG) spearheaded development of a short set (WG-SS) and later an extended set of disability indicators and the Child Functioning Module (CFM) for use in censuses and surveys. While use of these different instruments has increased nationally and internationally, it is necessary to scrutinize experiences gained to confirm strengths and reveal possible weaknesses and thus contribute to further development towards high-quality and comparable national and international disability statistics. This is of particular importance for monitoring implementation of the UN Convention on the Rights of Persons with Disabilities (CRPD), the disability specific indicators of the Sustainable Development Goals (SDGs) and other international initiatives that intend to improve the situation for more than one billion persons living with disabilities globally. In education systems it is important to develop reliable measures for the identification of children at risk of experiencing disability in order to enable adaptation of the learning environment to reach equitable conditions for learning and school achievement among children across different levels of functioning. This paper investigates experiences with using the CFM in school-based surveys in Niger and Ghana which provides a unique opportunity to analyze the agreement of disability measurement over time and between different respondents.

The development of CFM was initiated to address limitations in use of WG-SS among children. The main purpose of CFM is to enable identification of children with functional difficulties that may place children at risk of being disabled [3]. The six functional domains in WG-SS, i.e., seeing, hearing, walking, remembering and concentrating, communicating and self-care, were found to fall short of capturing the inherent complexity in determining child disability [4]. Therefore, new dimensions were included to capture child disability more fully; emotions, behavior, attention/concentrating, and coping with change [3].

It is well-known from the literature that disability may be fluctuating as individuals with disabilities at one point in time may not qualify as being disabled at a later point in time, and vice versa [5,6]. Individual experience of functional limitations can be seen as the result of an interaction between a person’s health status and both environmental and contextual factors [1]. Functional limitations may then vary with time due to changes in interactive factors, for instance medical and technological progress and improved access to services [7]. For instance, Krueger and Skoog [8] reported from a survey in the US that 40.5% of census respondents who indicated disability did not report any disability one year later. Using a large longitudinal database in Denmark using two different measurements including Washington Group Short Set, Amylon and Christensen [9] reported that the transition in and out of disability status was more common than enduring disability. Myers et al. [10] found that inconsistent answers to disability questions over time reflect real health-status changes. Impairments may thus have a high degree of variability over time and depending on individual and other surrounding factors, individuals may fluctuate in or out of disability [11,12]. Transitory disability may be explained in view of disability models such as ICF that understands disability as an interaction of impairment, health, individual and environmental factors. Changes in one or more factors may alter individuals’ activity limitations and/or restrictions in social participation and thus disability status.

A joint statement was published in 2017 by multiple UN organizations, member states, organizations of disabled people (OPDs) and others recommending the module as appropriate for SDG data aggregation for children [13,14]. This led to the roll-out of the CFM module as part of the Multiple Cluster Indicator Survey (MICS) (https://mics.unicef.org/, accessed on 4 June 2026). The module has been validated in different contexts around the world and is increasingly reported in the international literature. Further validation and adaptations have for instance taken place in Canada [15] and Uganda [16], both confirming the validity and applicability of CFM among caregivers as proxy respondents and children respectively. A validation study in Fiji confirmed the good diagnostic accuracy of CFM and a high inter-rater reliability between teacher and parent respondents concerning seeing, hearing and walking domains [17] but low to moderate accuracy for remembering and concentrating. In Finland, Ng et al. [18] found moderate to substantial intra-rater test–retest reliability of a modified CFM in a study among young adolescents completing the CFM twice with a two-week interval. UNICEF recently released a first batch of results from using the module in the Multiple Indicator Cluster Surveys (MICSs), showing that CFM produces higher estimates of childhood disability prevalence than the WG Short Set. The results from Serbia further revealed higher disability prevalence in disadvantaged communities [19]. Further research is necessary in different contexts, particularly on the statistical properties of CFM, to complement published results and to guide the use, utilization of results and future development of CFM.

The study Education outcome variability in children with disabilities: Structure, Institution or Agency? was carried out in Ghana and Niger from 2020 to 2024 by four collaborating research institutions: Fafo Institute of Labour and Social Research and Sintef from Norway, École Normale Supérieure (ENS) de l’Université Abdou-Moumouni in Niger and Kwame Nkrumah University of Science and Technology (KNUST) in Ghana. The purpose of the study was to improve our understanding of the factors that affect the enrolment, attendance, results and achievements of children with disabilities in mainstream primary schools in Ghana and Niger. The study comprises a comprehensive qualitative study, a mapping exercise among teachers, two surveys among teachers and two surveys among parents/households (baseline and follow-up). This paper investigates the stability and reliability of CFM over time and across different respondents.

## 2. Materials and Methods

The sampling frame for the study was inclusive of primary schools with registered children with disabilities in the Ashanti region in Ghana and Niamey in Niger. Both countries have a school system with six years of primary school (Grade 1–6) and enrolment in Grade 1 at age six. Inclusive schools are educational institutions that support and accommodate all students, including children with disabilities. From a comprehensive list of schools within the two regions, schools with a high number of children with disabilities were selected, considering geographic representation and school type (private/public), totalling 27 schools in Ghana and 18 schools in Niger.

Five data collections were carried out, of which four included the Child Functioning Module (households/parents) or the Child Functioning Module—Teacher Version (teachers). The difference between the two versions lies in the use of “student” in the questions in the teacher version, while “child” is used in the household version. Additionally, the household version includes a question about self-care.

One mapping exercise and four survey data collections were carried out. Data collection was paper-based in Niger and by means of tablets in Ghana. In both countries, testing for comprehension was carried out among both teachers and households prior to the main data collection. Training was also provided prior to data collection with weight on how to use CFM among both teachers and research assistants. In Ghana, household data collection was carried out by 18 research assistants and four supervisors organized in small teams. In Niger, 18 research assistants were deployed individually and were supported by two inspectors. A quality check of completed data was integrated in the tablet program in Ghana, while supervised completion was used in Niger. Although three of the four data collections at T1 and T2 took up to several months, the bulk of the data collection took place within more concentrated time periods. Table 1 illustrates the different data collections. 

1. Mapping by teachers (Niger: November–December 2021. Ghana: January 2022). (T0_Teachers_): all teachers for Grades 1, 3 and 5 in schools selected from the list of inclusive schools with registered children with disabilities filled out the CFM forms (5–17 years) for all children in their classes on their own, following a short training exercise. Mapping continued by adding new schools until the target of 300 children with disabilities was reached. For each child with a disability according to our definition, one child without disabilities was randomly selected from the same class. Following this procedure, 621 children were selected to participate in further data collection in Niger, while the corresponding figure for Ghana was 605. Of these, completion of questionnaires by both teachers and households included 387 children in Ghana and 573 children in Niger. The reason for the lower figure in Ghana was much higher rejection of consent and assent. Small variations in N in the analyses (Supplementary Files) are due to several reasons such as teachers and/or children moving or being difficult to find and questionnaires being discarded.

2. Two rounds of questionnaire-based interviews with teachers (T1_Teachers_ and T2_Teachers_) (T1: Niger: February–May 2022. Ghana: January–July 2022, T2: Niger: May 2023. Ghana: March–April 2023) who were involved in the initial mapping and with parents/households of children identified as being disabled and the control children without disabilities (T1_Households_ and T2_Households_). CFM was not included in the first survey among teachers at T1 due to its proximity in time to the mapping, and the mapping results from using the CFM (T0_Teachers_) were used as part of T1_Teachers_. Teachers were interviewed in school mainly during the break during school time. Parents were interviewed either in their homes or at their children’s school. Children were in Grades 1, 3 and 5 in the first two data collections (teachers and households) and in Grades 2, 4 and 6 in the second data collection one year later.

The analyses of responses to the CFM questions used in this paper are from four of the five data collections: teacher mapping (T0_Teachers_), the second teacher survey (T2_Teachers_), and the two household surveys (T1_Households_ and T2_Households_). The surveys obtained approval from SIKT in Norway (Norwegian Agency for Shared Services in Education and Research), which conducted the Data Protection Impact Assessment (DPIA), with project number 819931. In Niger, ethical approval was granted by the research directorate in the Ministry of Higher Education and Research. In Ghana, the Ashanti Regional Director of Education and the Committee for Human Research, Publication and Ethics of the Kwame Nkrumah University of Science and Technology, School of Medical Sciences, and Komfo Anokye Teaching Hospital (reference number CHRPE/AP/001/22) granted the study ethical approval. Prior to data collection, participants were given verbal and written information about the surveys and their consent was obtained.

Coding followed the standard WG/UNICEF cut-offs: severe disability was defined as at least one “Cannot do at all” or “A lot of difficulty” on any of the functional domains. Moderate disability is defined as at least one “some difficulty” on any of the physical or sensory domains, i.e., including only the first six domains in the CFM (Box 1). The decision to deviate on this point from the broader CFM framework was due to high/very high scores on several of the cognitive, behavioral and attention-related domains—and thus helped to avoid inflated disability prevalence in the study population. Zhang and Holden [20] found the same in using MICS data from eight African countries and support the procedure chosen in our study. This may also have contributed to increased agreement between assessments. Descriptive statistics comprising frequency and percentages as well as estimated prevalence with (i) sensory and physical domains and (ii) all domains as the operational definition of disability are presented in the Supplementary Tables.

Box 1 **Child Functioning Module (CFM) used in the survey (version for teachers).**

AD1 Does this student wear glasses or contact lenses?^1^

CF1 If yes: when wearing his/her glasses/lenses, does this student have difficulty seeing?^2^

    If no: does this student have difficulty seeing?^2^

AD2 Does this student use a hearing aid?^1^

CF2 If yes: when using his/her hearing aid, does this student have difficulty hearing sounds like people’s voices or music?^2^

    If no: does this student have difficulty hearing sounds like people’s voices or music?^2^

AD3 Does this student use any equipment or receive assistance for walking?^1^

CF3 If yes: without the use of his/her equipment or assistance, does this student have difficulty walking?

    If no: does this student have difficulty walking?^2^

CF4 When this student speaks, does he/she have difficulty being understood by you or others in this classroom?^2^

CF5 Compared with children of the same age, does this student have difficulty learning things?^2^

CF6 Compared with children of the same age, does this student have difficulty remembering things?^2^

CF7 Does this student have difficulty concentrating on an activity that he/she enjoys doing?^2^

CF8 Does this student have difficulty accepting change in his/her routine?^2^

CF9 Compared with children of the same age, does this student have difficulty controlling his/her behaviour?^2^

CF10 Does this student have difficulty making friends?^2^

CF11 How often does this student seem very anxious, nervous or worried?^3^

CF12 How often does this student seem very sad or depressed?^3^

Answer categories: ^1^ 1 = Yes, 2 = No, ^2^ 1 = No difficulty, 2 = Some difficulty, 3 = A lot of difficulty, 4 = Cannot do at all, ^3^ 1 = Daily, 2 = Weekly, 3 = Monthly, 4 = A few times a year, 5 = Never.

## 3. Results

Table 2 below shows the % of correspondence across the different data collections and respondents (teachers and parents/family) for each country separately. This provides insight into the stability of disability measures over time with largely the same respondents and correspondence between two different groups of respondents (teachers and parents). Inter-rater agreement/reliability is calculated as the percentage of corresponding measures across two data collections with different respondents (teachers and parents). Test–retest reliability is estimated when comparing changes over time with the same respondents (teacherT0–teacherT2, parentsT1–parentsT2). Due to the one-year time lap between the two waves of data collection in our study, several factors such as children’s development, grade progression, changes in learning environments, shifts in parents’ and teachers’ perceptions, as well as possible variations in circumstances around data collection invite a cautious interpretation of this not as a strict test–retest reliability study but rather as an assessment of classification consistency. Table 2 shows that there are small differences between the two country samples, and the overall patterns are similar when comparing results from the two countries. 

In Ghana, no severe disability was reported in all four data collections for 137 (35.4%) children. For 139 (35.9%) children, only one out of four data collections identified a child as being severely disabled. The corresponding figures for two out of four, three out of four and all four were 68 (17.6%), 30 (7.8%) and 13 (3.4%) responses. Complete match among the four assessments (severe disability) was 38.8% (all four data collections give the same result for 137 + 13 = 150 children). Corresponding figures for moderate disability show somewhat lower correspondence between respondents, with 30.7% (24.8% + 5.9%) showing a complete match (all four data collections give the same result for 96 + 23 = 119 children).

In Niger, no severe disability was reported in all four data collections for 198 (34.6%) children. For 191 (33.3%) children, only one out of four data collections identified a child as being severely disabled. The corresponding figures for two out of four, three out of four and all four were 114 (19.9%), 48 (8.4%) and 22 (3.8%) responses. The percentage of complete matches found among the four assessments (severe disability) was 38.4% (34.6% + 3.8%) (all four data collections give the same result for 198 + 22 = 220 children). Corresponding figures for moderate disability show somewhat lower correspondence between respondents, reaching 29.3% (all four data collections give the same result for 130 + 39 = 169 children).

The observable agreement for Table 2 was 0.55 and the Fleiss kappa was 0.17, implying slight to fair agreement [21].

**Figure 1:**
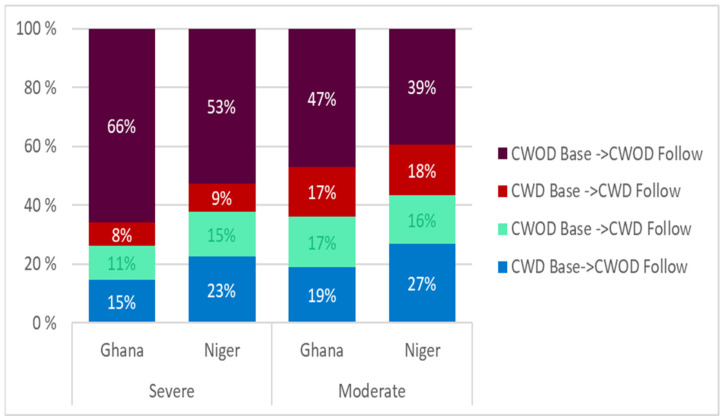
Agreement in households’ disability assessment at baseline and follow-up (T_1HHs_–T_2HHS_).

Figure 1 shows agreement in assessments by households over time (teacher and household baseline and follow-up). For the severe disability threshold, 66% of the children in Ghana were assessed as not having a disability (child without disability—CWOD) both at baseline and follow-up, with the corresponding figure for Niger being 53%. Agreement of being assessed as a child with a disability (CWD) at both times was 8% in Ghana and 9% in Niger. Disagreement in assessment reached 11% in Ghana for children being assessed as not having a disability at baseline and as having a disability at follow-up, with the corresponding figure for Niger being 15%. The percentage of children assessed as having a disability at baseline and as not having a disability at follow-up reached 15% in Ghana and 23% in Niger.

When lowering the threshold for being disabled to at least some difficulty (moderate disability), 47% and 39% of the children in Ghana and Niger respectively were assessed as not having a disability both at baseline and follow-up. The percentage of children being assessed as having a disability at both baseline and follow-up was 17% in Ghana and 18% in Niger. Disagreement in assessment reached 17% in Ghana and 18% in Niger when looking at the shift from CWOD to CWD, and 19% in Ghana and 27% in Niger for the shift from CWD to CWOD. Agreement between assessments is higher in Ghana than in Niger for both disability thresholds.

**Figure 2:**
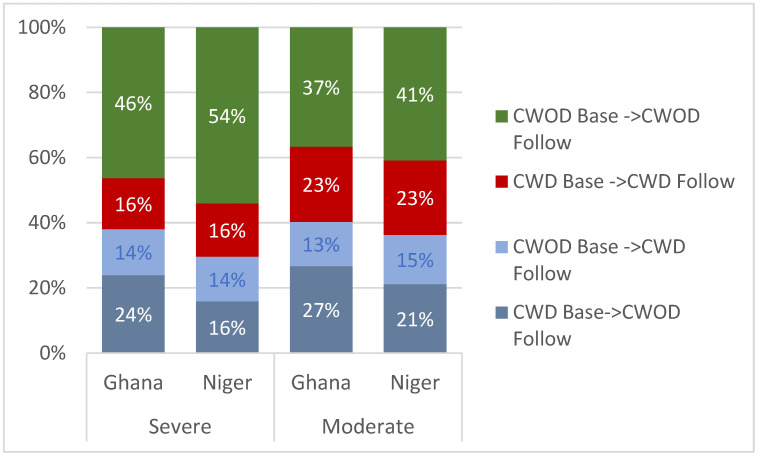
Agreement in teachers’ disability assessment at baseline and follow-up (T0_Teachers_–T2_Teachers)_.

Figure 2 shows agreement in assessments by teachers over time (mapping and follow-up). For the severe disability threshold, 46% of the children in Ghana were assessed as not having a disability both at baseline and follow-up, with the corresponding figure for Niger being 54%. Agreement of being assessed as a child with a disability at both times was 16% both in Ghana and Niger. Disagreement in assessment was 14% both in Ghana and Niger for children being assessed as being without a disability at baseline and with a disability at follow-up. The percentage of children assessed with a disability at baseline and without a disability at follow-up reached 24% in Ghana and 16% in Niger.

When lowering the threshold for being disabled to at least some difficulty (moderate disability), 37% and 41% of the children in Ghana and Niger respectively were assessed as not having a disability both at baseline and follow-up. The percentage of children being assessed as having a disability at both baseline and follow-up was 23% both in Ghana and Niger. Disagreement in assessment reached 27% in Ghana and 21% in Niger when looking at the shift from CWOD to CWD, and 27% in Ghana and 21% in Niger for the shift from CWD to CWOD. For teachers, agreement between assessments is higher in Niger than in Ghana for both disability thresholds.

**Figure 3:**
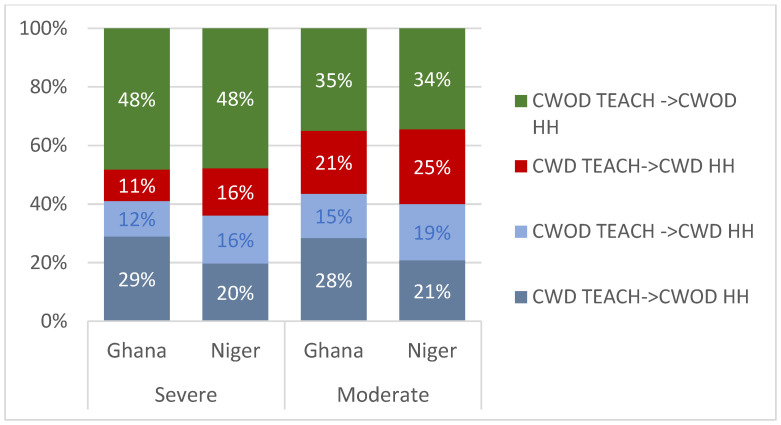
Agreement between teachers’ and households’ disability assessments at baseline (T0_Teachers_–T1_HHs)_.

Figure 3 shows agreement in assessment at baseline by teachers and households. For the severe disability threshold, 48% in both Ghana and in Niger were assessed by both teachers and households as not having a disability. The percentage assessed as having a disability by both teachers and households was 11% in Ghana and 16% in Niger. Looking at disagreement in assessment, for children assessed as being severely disabled, 12% and 16% in Ghana and Niger respectively were assessed by their teacher as not being disabled and as disabled by their households. Corresponding figures for teachers assessing the children as being disabled while households assessed the children as being without disabilities were 29% and 20% for Ghana and Niger respectively.

For the lower threshold (moderate disability = at least some difficulty), 35% in Ghana and 34% in Niger were assessed by both teachers and households as being without a disability. The corresponding figures for having a disability were 21% and 25%. Concerning disagreement, 15% in Ghana and 19% in Niger were assessed by their teacher as not being disabled while households assessed them as being disabled. Finally, in Ghana, 28% were assessed by their teachers as being disabled at baseline but without a disability by their households. The corresponding result from Niger was 21%.

**Figure 4:**
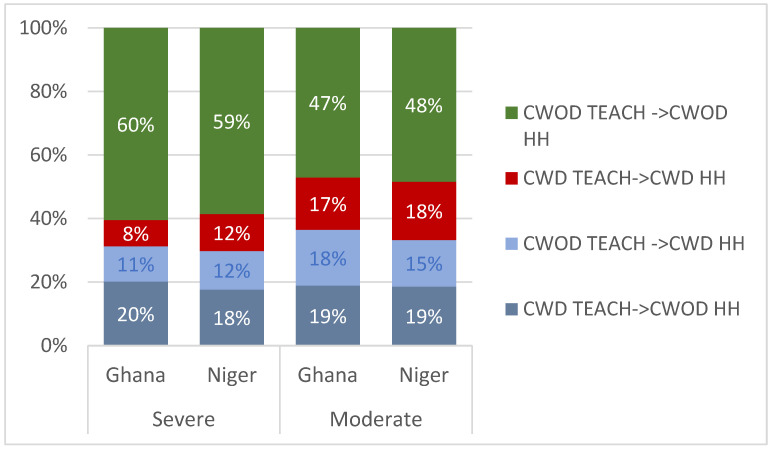
Agreement between teachers’ and households’ disability assessments at follow-up (T2_Teachers_–T2_HHs)_.

Figure 4 shows a comparison of teachers’ and households’ assessments at follow-up. For severe disability, 60% in Ghana and 59% in Niger are assessed as being without a disability, while corresponding figures for being with a disability are 8% and 12%. Disagreement in teachers assessing children as not having disabilities and households assessing children as having disabilities reaches 11% in Ghana and 12% in Niger. For the other disagreement, i.e., teachers’ assessment of children to have a disability and households’ assessment of children to not have a disability, figures are 20% for Ghana and 18% for Niger.

For at least some difficulty (moderate disability), 47% in Ghana and 48% in Niger are assessed as not having a disability by both teachers and households. Corresponding figures for having a disability are 17% and 18%. Concerning disagreement, 18% in Ghana and 15% in Niger are assessed by their teachers as being without a disability and as disabled by their households. In both countries 19% are assessed as having a disability by their teachers but not by their households.

Assessment agreements shown in Table 3 and Table 4 are drawn from the tables in Supplementary File S2 and calculated as the percentage of children who were assessed as having a disability in both assessments that are compared + children who were assessed as not having a disability in both assessments. Firstly, Table 3 and Table 4 shows that there is moderate variation between the different agreements, varying from a high of 73.9% (household baseline and follow-up among severely disabled children in Ghana) to a low of 56.5% (baseline mapping and household among moderately disabled children in Ghana). Secondly, no apparent systematic difference appears when looking at the different comparisons, with the exception that agreement tends to be higher for severely disabled children compared to those with moderate disability.

The reliability statistics presented in Table 3 and Table 4 should be interpreted in light of the prevalence paradox, that is, the skewed distributions in the data can lead to an underestimation of agreement. In contrast, the substantially higher PABAK (Prevalence-Adjusted and Bias-Adjusted Kappa) values suggest that the level of agreement between raters is stronger than that indicated by Cohen’s kappa. To avoid misinterpretation, both statistics are reported and can be considered jointly in the assessment of agreement. Table 3 and Table 4 shows that Cohen’s kappa is low, but PABAK is considerably higher, adjusting for the prevalence paradox.

Assessment agreement was also analyzed by sub-groups. No systematic differences in agreement were found between boys and girls, while analyses revealed a weak tendency towards higher agreement in rural vs urban areas (analyses in Supplementary File S2) and similarly that agreement tended towards being higher among Grade 5 students.

## 4. Discussion

The study compared teachers and household respondents’ abilities to identify children as disabled (according to WG guidelines) using the CFM in one mapping exercise and in two rounds of surveys over time among school children in Ghana and Niger. Observed agreement among and between teachers and households appears to be relatively stable, varying largely between 60 and 70%, and is slightly higher using the severe disability threshold. This means that there is an observed discrepancy in disability status assessment of 30–40% over time and between respondent groups. Adjusted kappa statistics reveal improved agreement both over time with the same group of respondents and between different respondents. While Cohen’s kappa suggests lower than observed agreement, PABAK adjusts for unequal category prevalence and systematic rater bias, suggesting a somewhat more favorable interpretation.

Comparing results from the teacher mapping, two household surveys and the second teacher survey reveal that, with some variation, somewhat below two thirds obtain the same disability status across data collections. Mostly this is children without disabilities. For the remaining, where approximately one third have some variation, there is a combination of transcending from being disabled to being non-disabled and vice versa. A somewhat better correspondence among severely disabled is as expected due to the higher threshold. We can assume a higher proportion of visible impairments among children who were assessed as being severely disabled. Moderate disability status may on the other hand for many not be visible, and it may be more difficult to observe and more transitional, which may then result in more variation in the assessments.

Without an agreed standard, it is a matter of discussion whether the demonstrated level of stability over time with the same group of respondents and agreement across different respondents is acceptable. A total of 60–70% agreement needs to be considered in view of methodological issues with the study that is highlighted below, with additional caution due to the reliability statistics, but also in view of assessment of functioning being context-specific. Multiple proxies will capture genuinely different aspects of functioning. Teachers have a professional background, relate to and know the children within the specific context of the classroom, their role as teachers and the school environment and carried out the assessment within this context. Parents obviously have a different role, know the children from a very different context, will have mixed background and mostly carried out the assessment in the confinement of their own home. Expected transitioning in or out of disability over time between data collections, which in our case was close to one year, helps to explain the disagreement in assessment over time. The rather low correspondence between the four data collections as shown in Table 2 adds further limitations to the observed results and indicates both that assessments based on CFM and similar instruments come with a level of uncertainty and should ideally be complemented with multiple respondents. On the other hand, it is important to underline that the purpose of CFM is to assess the risk of disability in larger surveys and not to replace clinical instruments. Bearing in mind the practical implications of including multiple respondents, such as time, costs and complexity of organizing the study, the advantage of using multiple respondents will necessarily need to be balanced against these and other factors. This includes also the purpose of the data collection and multiple respondents may for instance be less critical in national surveys than in school-based surveys that can impact directly on the learning environment for children with disabilities. It may further be that using multiple respondents adds more value for the less visible cognitive and emotional domains of CFM.

Bearing in mind both the time gap, the different character of the initial assessment by teachers and that there are two different groups of respondents, less correspondence could possibly be expected. This is particularly so for children due to large variations in growth and development [4] and changes in underlying health status [10] as well as variation in external factors that may influence disability status [11]. A child may fall behind for instance in social behavior at one stage and then catch up with his/her peers over time, either due to their slow pace of development or because their learning environment has changed for the better [12].

### Strengths and Weaknesses

The main strength of this study is that it has been possible to compare assessments of the same group of children over time and between two groups of respondents (teachers and parents). This is a more advanced design than most relevant disability surveys in LMICs. The results of the study have given new insights into the quality of CFM and thus contribute to the field of disability statistics.

This study relies on and compares teachers’ and parents’ perceptions of the same children. It is not stated that one has more insight into children’s functioning than the other, and the basis for the assessment differs. Their perspectives complement each other but are not complete. Additionally, possible differences in the instructions given to both teachers and households are not known and may have had some impact on responses in the two countries. It is further a possibility that differences in the method of data collection, i.e., paper-based vs. tablet-based, have some influence on the responses. Another uncertainty lies in possible differences in understanding of the answer categories (“A lot of difficulty”, “Cannot do”). This was discovered during the quality check of teachers’ responses and led to some adjustments in inclusion criteria. All these factors may have had some impact on the results from comparing teachers’ and parents’ assessments in the study, although this is less likely to concern the patterns that have been demonstrated.

Due to the way sampling was carried out, the study is not representative for schools in the region and cannot be interpreted as national or regional prevalence estimates, but as showing agreement and classification stability within a purposively selected school-based sample.

Real changes in disability status of children over time may have affected comparison in the study and thus have some impact on the results. Transition into or out of being disabled occurs and will affect measurement agreement over time. This partly explains some of the variations observed, although we do not know how much it contributes. Another factor that may explain some of the disagreement is the possibility of a “teaching effect” from repeating surveys, i.e., for instance increased awareness can counter misconceptions or chang the understanding of disability. These and other factors will affect assessments regardless of the design and they are not a particular weakness of this study. Finally, the choice to use only the physical and sensory domains in the CFM to define “moderate disability” to reduce inflated prevalence of disability may have contributed to increased agreement due to larger variation in responses to cognitive, behavioral and attention-related domains.

## 5. Conclusions

The study has demonstrated observed CFM assessment consistency of 60–70% over time and between two different respondent groups in two countries, using CFM. Further analyses provided reason for caution when interpreting observed results and that consistency in reality is lower than observed agreement. Bearing in mind the several reasons that can explain the disagreement in assessment, the results still indicate that CFM is useful as a survey instrument for identifying patterns of functional difficulties in children at the group or population level. CFM is practical in use and can easily be integrated in larger population studies and thus can contribute to bringing disability into mainstream public health initiatives.

One takeout from this study may be that there is a need for multiple respondents in reporting disability status when proxy reporting is used, bearing in mind that this needs to be balanced against both the purpose of studies and practical realities such as time and costs. Further studies are needed in different contexts to reach a stronger evidence base on disability agreement/disagreement among children and adolescents when using the CFM.

## Figures and Tables

**Table 1:** Timing of data collections.

Year	2021	2022	2023
Data collections	Selected from mapping		
Teachers	T0_Teachers_	T1_Teachers_	T2_Teachers_
N_Ghana_/N_Niger_	4214/5194	605/621	399/430
Households		T1_HHS_	T2_HHS_
N_Ghana_/N_Niger_		410/581	387/573

**Table 2:** Correspondence between four data collections (T1_HHs_, T0_Teachers_, T2_HHs_, T2_Teachers_).

Number of Data Collections (Out of Four) Reporting the Child as Disabled	Frequency (%) of at Least Moderate Disability		Frequency (%) of at Least Severe Disability	
	Ghana	Niger	Ghana	Niger
0 (data collections)	24.8	22.7	35.4	34.6
1	27.1	30.9	35.9	34.3
2	25.3	26.0	17.6	19.9
3	16.8	13.6	7.8	8.4
4	5.9	6.8	3.4	3.8
	100.0	100.0	100.0	100.0

**Table 3:** Agreement of assessment of children with moderate and severe disability at T1_HHS_/T0_Teacher_ and T2 in Niger.

Compared Data Collections	At Both Times		Observed Agreement	Cohen’s Kappa	95% CI Low/High	PABAK
	% Not Disabled	% Disabled				
Severe disability						
T1_HHS_–T2_HHS_	52.7	9.4	62.1	0.074	−0.009/0.156	0.242
T0_Teachers_–T2_Teachers_	54.0	16.4	70.4	0.306	0.204/0.402	0.408
T0_Teachers_–T1_HHS_	47.2	16.2	63.4	0.190	0.103/0.270	0.268
T2_Teachers_–T2_HHS_	58.6	11.6	70.2	0.239	0.137/0.335	0.404
Moderate disability						
T1_HHS_–T2_HHS_	39.3	17.4	56.7	0.101	0.021/0.179	0.134
T0_Teachers_–T2_Teachers_	41.0	22.7	63.7	0.249	0.155/0.346	0.274
T0_Teachers_–T1_HHS_	34.3	25.1	59.4	0.181	0.102/0.262	0.188
T2_Teachers_–T2_HHS_	48.4	18.3	66.7	0.270	0.178/0.362	0.334

**Table 4:** Agreement of assessment of children with moderate and severe disability at T1_HHS_/T0_Teacher_ and T2 in Ghana.

Compared Data Collections	At Both Times		Observed Agreement	Cohen’s Kappa	95% CI Low/High	PABAK
	% Not Disabled	% Disabled				
Severe disability						
T1_HHS_–T2_HHS_	65.9	8.0	73.9	0.216	0.109/0.323	0.478
T0_Teachers_–T2_Teachers_	46.4	14.8	61.2	0.146	0.048/0.247	0.224
T0_Teachers_–T1_HHS_	50.2	9.8	60.0	0.074	−0.020/0.161	0.200
T2_Teachers_–T2_HHS_	60.5	8.2	68.7	0.150	0.050/0.258	0.374
Moderate disability						
T1_HHS_–T2_HHS_	47.0	16.8	63.8	0.204	0.104/0.302	0.276
T0_Teachers_–T2_Teachers_	36.8	22.1	58.9	0.175	0.081/0.268	0.178
T0_Teachers_–T1_HHS_	37.1	19.8	56.9	0.127	0.031/0.222	0.138
T2_Teachers_–T2_HHS_	47.0	16.6	63.6	0.197	0.101/0.296	0.272

## Data Availability

The original contributions presented in this study are included in the article and Supplementary Materials. Further inquiries can be directed to the corresponding author.

## Supplementary Materials

The following supporting information can be downloaded at: https://www.mdpi.com/article/10.3390/ijerph23070892/s1, File S1: Prevalence estimates and agreement; File S2: Frequencies. Tables include data on frequencies and percentages of the CFM domains and data which is the basis for the calculation of agreement.

## Abbreviations

The following abbreviations are used in this manuscript:

WG	Washington Group on Disability Statistics
WG-SS	Washington Group Short Set CRPD
CFM	Washington Group/UNICEF Child Functioning Module
ICF	International Classification of Functioning, Disability and Health
CRPD	United Nations Convention on the Rights of People with Disabilities
SDGs	Sustainable Development Goals
OPDs	Organizations of Disabled People
MICS	Multiple Indicator Cluster Surveys
ENS	École Normale Supérieure
KNUST	Kwame Nkrumah University of Science and Technology
